# Retraction Note: ‘Self-stigma’ of people with cutaneous leishmaniasis the unrecognized one: what do we think; what do we know; what can we prove?

**DOI:** 10.1186/s12939-024-02108-4

**Published:** 2024-03-19

**Authors:** Binega Hagos, Zenawi Zerihun

**Affiliations:** 1 Department of Social work, Mekelle University, Tigrai, Mekelle, Ethiopia; 2 Department of Psychology, Mekelle University, Tigrai, Mekelle, Ethiopia

**Retraction Note: International Journal for Equity in Health (2023) 22:180**

10.1186/s12939-023-01998-0

The authors have retracted this article because they did not have permission to publish the data presented. The scientific content has been removed at the request of the data owner. Both authors agree with this retraction.

